# Age Inclusive Management Strategies: Applying a Public Policy Framework to Institutions of Higher Education

**DOI:** 10.1093/geroni/igaf122.1273

**Published:** 2025-12-31

**Authors:** Brian Kaskie

**Affiliations:** University of Iowa, Iowa City, Iowa, United States

## Abstract

The Age Inclusive Management Strategies Advisory Council, established in 2024, comprised 20 representatives from diverse employment sectors including two institutions of higher education. These employment leaders offered their perspectives about the best ways to recruit, retain and promote workplace inclusivity for aging employees. Quarterly meetings and workgroups identified actionable policy alternatives, focusing on the Workforce Innovation and Opportunity Act (WIOA), the Senior Community Service Employment Program (SCSEP), caregiving policies, and age discrimination. The Council built this agenda upon foundational research from the AIMS initiative which identified gaps in current employer practices relative to need and demand among aging employees and surveyed employer awareness of workforce policies and programs designed for employees over 50. The council identified several alternatives to address the recruitment and retention of an aging workforce, aligning each with an established policy platform operating within state agencies including aging, labor, human rights, and the governor’s office. Relative to the aging workforce within institutions of higher education, we highlight how federal and state programs support employment of older workers through skills training and job placement programs. We then examine how institutions of higher education have adopted such age inclusive management strategies and focus on how caregiving challenges faced by older workers have been addressed through employee assistance programs and other supportive services. Supporting the employment of older persons who are able and want to work corresponds with several benefits. Employers address labor force needs and older adults retain income and benefits that promote financial security, health, and successful aging.

